# Critical Factors in Sample Collection and Preparation for Clinical Metabolomics of Underexplored Biological Specimens

**DOI:** 10.3390/metabo14010036

**Published:** 2024-01-05

**Authors:** Hygor M. R. de Souza, Tássia T. P. Pereira, Hanna C. de Sá, Marina A. Alves, Rafael Garrett, Gisele A. B. Canuto

**Affiliations:** 1 Instituto de Química, Universidade Federal do Rio de Janeiro, LabMeta—LADETEC, Rio de Janeiro 21941-598, Brazil; ribeirohygor@ufrj.br; 2 Departamento de Genética, Ecologia e Evolucao, Universidade Federal de Minas Gerais, Belo Horizonte 31270-901, Brazil; tassia.pontes@ufop.edu.br; 3 Departamento de Biodiversidade, Evolução e Meio Ambiente, Universidade Federal de Ouro Preto, Ouro Preto 35400-000, Brazil; 4 Departamento de Química Analítica, Instituto de Química, Universidade Federal da Bahia, Salvador 40170-115, Brazil; carvalho.hanna@ufba.br; 5 Instituto de Pesquisa de Produtos Naturais Walter Mors, Universidade Federal do Rio de Janeiro, Rio de Janeiro 21941-599, Brazil; marina.amaral@ippn.ufrj.br; 6 Department of Laboratory Medicine, Boston Children’s Hospital—Harvard Medical School, Boston, MA 02115, USA

**Keywords:** biological specimens, pre-analytical factors, non-invasive collection, metabolite extraction, mass spectrometry, biomarkers, metabolomics

## Abstract

This review article compiles critical pre-analytical factors for sample collection and extraction of eight uncommon or underexplored biological specimens (human breast milk, ocular fluids, sebum, seminal plasma, sweat, hair, saliva, and cerebrospinal fluid) under the perspective of clinical metabolomics. These samples are interesting for metabolomics studies as they reflect the status of living organisms and can be applied for diagnostic purposes and biomarker discovery. Pre-collection and collection procedures are critical, requiring protocols to be standardized to avoid contamination and bias. Such procedures must consider cleaning the collection area, sample stimulation, diet, and food and drug intake, among other factors that impact the lack of homogeneity of the sample group. Precipitation of proteins and removal of salts and cell debris are the most used sample preparation procedures. This review intends to provide a global view of the practical aspects that most impact results, serving as a starting point for the designing of metabolomic experiments.

## 1. Introduction

Metabolomics is a field of study aiming to comprehensively investigate metabolites present in a biological system and their interactions with other molecules. It involves the systematic identification and quantification of small molecules, generally described as those below 1500 Da, providing insights into biological processes within a system [1]. It is a diverse and multidisciplinary field that is constantly evolving, with new methods and applications often described.

Sample collection and preparation are crucial steps in any metabolomics study design, profoundly impacting the coverage and quality of the data obtained through instrumental analyses [2,3,4]. Nowadays, a myriad of biological specimens are being used for metabolomic investigations, some very usual, such as plasma, serum, and urine; and others uncommon or less explored, such as tears, seminal fluid, sweat, and hair [5,6,7].

Improper sample collection and storage can lead to metabolite alteration, transformation, and degradation, resulting in inaccurate interpretation of a system’s biochemical processes. Regardless of the sample matrix, care should be taken to preserve the sample integrity during and after collection by stopping any enzymatic activity, typically via rapidly freezing combined or not combined with organic solvent addition [3,4]. Similarly, an inadequate sample preparation protocol for a particular biological question can lead to metabolome coverage outside the scope of the project and, consequently, meaningless results and frustration. Standardized protocols for sample preparation are essential to isolate metabolites from biological matrices, obtaining reliable and high-quality results. The ultimate goal is to maximize metabolite coverage while minimizing the matrix effect and metabolite degradation. Overall, when working with untargeted metabolomics, one should consider a method that is non-selective, simple yet robust, and reproducible [8].

Mass spectrometry (MS) is the most used analytical technique in metabolomics. Combined with liquid or gas chromatography, it allows the separation, identification, and quantification of a broad range of metabolites, ranging from nonpolar to polar compounds. Its key advantages compared to other techniques (for instance, nuclear magnetic resonance or infrared spectroscopy) are its high sensitivity, specificity, and high throughput analysis, allowing for the detection, differentiation, and identification of low-abundance metabolites with high confidence [9]. In addition, because of its versatile combination with several ionization probes, MS is used to analyze a wide range of biological specimens.

Sample preparation in metabolomics-based mass spectrometry analyses can be challenging when working with uncommon or less explored biological specimens since optimized and standardized protocols are scarce in the current literature. To address this issue, this review will discuss critical practical aspects of sample collection and preparation of eight biological specimens, other than blood (serum/plasma) and urine, employed in clinical metabolomics studies. This review is divided into two main sections: uncommon biological specimens in metabolomic investigations (human breast milk, ocular fluids, sebum, and seminal plasma) and specimens widely applied to toxicological and clinical practices (cerebrospinal fluid, hair, saliva, and sweat) and that can be better explored in metabolomics. These biological samples present a great potential for investigation through clinical metabolomics since they reflect the status of living organisms, with future applications in discovering biomarkers to diagnose different diseases. The discussions presented here may be a starting point for researchers who wish to work with these samples.

## 2. Literature Search and Review Outline

The literature search was performed on the PubMed database (http://www.ncbi.nlm.nih.gov/pubmed, accessed on 10 November 2023) querying full-text publications written in English over the last five years (2019–2023), combining the words “metabolomics” and “mass spectrometry” with each biological specimen’s name and synonyms. Only research articles applying liquid chromatography–mass spectrometry (LC–MS), gas chromatography–mass spectrometry (GC–MS), or direct infusion mass spectrometry (DIMS), including conductive polymer spray ionization MS (CPSI) and desorption electrospray ionization MS (DESI), were considered in the revision. 

**Figure 1:**
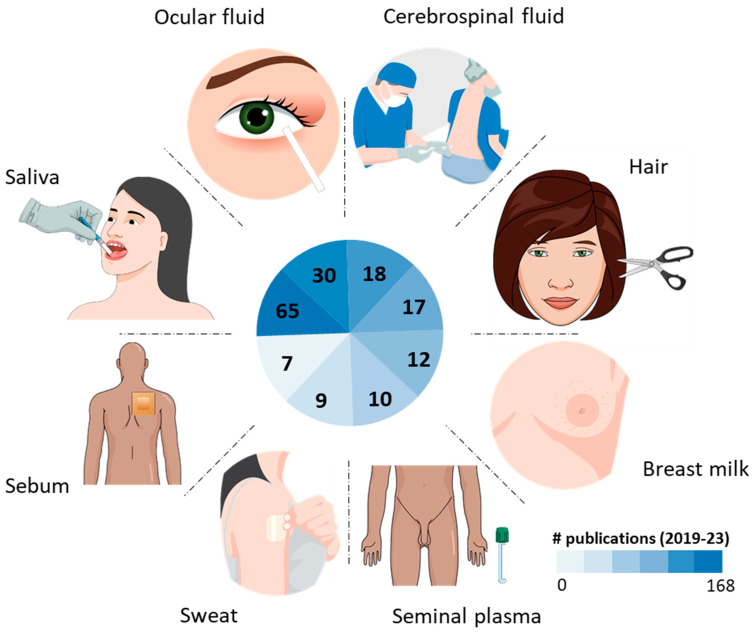
Distribution of publications on uncommon and underexplored biological specimens in clinical metabolomics using chromatography/mass spectrometry-based methods.

Our search led to an initial compilation of more than 200 papers, resulting in 168 final publications. Figure 1 presents the number of publications per biological specimen. Sample collection, pre-processing factors, and metabolite extraction procedures are detailed in Table S1 of the Supplementary Material. Table 1 highlights the pre-analytical factors that most impact metabolomic results for each specimen.

## 3. Uncommon Biological Specimens in Clinical Metabolomics

### 3.1. Human Breast Milk (HBM)

Human breast milk (HBM) is the most recommended food for newborns [10]. It is a complex fluid containing a wide range of substances produced by the mother organism and those introduced into the mother’s body through ingesting exogenous metabolites [11]. Breast milk is commonly divided into three categories, colostrum, transitional milk, and mature milk, which refer to the gradual change in the milk content throughout lactation [12]. Lipid species constitute a large part of the HBM. However, polar metabolites, such as oligosaccharides, amino sugars, creatine, carnitine, free amino acids, nucleic acids, nucleotides, and polyamines, are also HBM components required for proper child development [11]. The most common methods for HBM collection are by hand and through pump expression into bisphenol A-free polypropylene containers, in which breast skin can be cleaned with water and soap and the first drops discarded [13,14,15]. After collection, HBM is generally transported at 4 °C [16] and stored at −20 °C or −80 °C until analysis [17,18,19]. Some techniques, like pasteurization and rapid freezing, can be used to reduce potential microbial contamination before metabolite extraction. However, previous studies [14,20] have suggested that pasteurization affects the lipid and metabolite composition of human milk, posing nutritional consequences and pre-analytical issues.

Generally, the sample preparation strategy for the polar fraction of HBM is performed by adding cold (below 4 °C) organic–aqueous solvents (methanol and/or acetonitrile with water) followed by a centrifugation step [13,17,20,21,22]. Alternatively, some commonly used extraction procedures for lipid and nonpolar analyses include mixtures of methanol and chloroform (e.g., Bligh and Dyer—B&D—and Folch methods) [16,19]. Nevertheless, the extraction procedure with the less toxic methyl *tert*-butyl ether (MTBE) combined with methanol has been highly recommended for medium and nonpolar metabolites [14,15,18,23,24]. 

A dilution of the extract is required to avoid mass spectrometry signal saturation for high-abundance compounds, but metabolites present at low concentrations in HBM may not be detected. To avoid this limitation, Hewelt-Belka and coworkers recommend the combination of two extraction techniques: solid-phase extraction (SPE) and liquid–liquid extraction (LLE) [19]. This strategy can be performed in two steps. Based on SPE and employing a zirconia-based stationary phase, the first step enabled the selective isolation and enrichment of the low-abundance HBM lipid species. The extract obtained in the second step, based on LLE using the B&D method, is used as a dissolving solution for the enriched fraction obtained in the first step. This analytical approach allows extensive metabolome coverage, especially for low-abundance glycerophospholipids and sphingolipids.

### 3.2. Ocular Fluids

Metabolomics using ocular fluids offers a promising approach to elucidate the pathogenesis of ophthalmologic disorders and discover potential biomarkers that contribute to improving and developing new diagnostic and treatment methods. The main fluids used in such studies include tears, aqueous humor (AH), and vitreous humor (VH). Tears, also called tear film (TF), are composed of three layers: the lipid layer containing nonpolar and amphiphilic lipids; the aqueous layer composed of electrolytes, proteins, and metabolites such as amino acids, urea, and glucose; and the mucosal layer consisting of glycoproteins [25]. The aqueous humor is a clear fluid that fills the lower and upper chambers of the eyes. The AH is a complex mixture of water, electrolytes, proteins, and metabolites (such as glutathione, urea, and amino acids) [26]. The VH is the fluid that fills the space between the retina and lens and is composed of collagen, glycoproteins, salts, and carbohydrates [5].

Tears are collected through two simple and non-invasive methods: using an absorbent material, named Schirmer tear strips (TSs), and through capillarity. TS collection is performed by wiping tears by placing the strip on the lower eyelid for 5–10 min [27,28]. The capillary collection is performed using glass capillary tubes positioned in the conjunctival fornix. During the procedure, the patient is instructed to lower his head, and tears are collected through gravity. Catanese et al. [29] compared and validated these two collection methods for metabolomics analysis. The results showed a greater number of significant metabolites and less variability when using capillary collection. In addition, some disadvantages of TSs were also considered, such as the impossibility of quantifying the volume of tears and the increased risk of contamination due to the direct contact of the collection strip with the ocular surface. Therefore, disinfecting the area and waiting at least two hours after awakening should be considered before tear collection [30]. Unlike tears, aqueous and vitreous humor are collected invasively using surgical procedures, which require the application of local anesthesia and disinfection of the ocular surface. AH collection is performed through a paracentesis procedure, in which a needle is used to aspirate the fluid. The VH is collected through vitrectomy, in which the vitreous humor is cut and aspirated [31,32]. After collection, all ocular fluids are immediately stored at −80 °C until analysis.

The extraction procedure of tears is determined according to the type of collection. When TSs are used, the extraction is performed using a mechanical device, such as a bench mill. The strips are cut, and the combination of organic solvents and the shock of the beads against the strips promotes rapid extraction. Cicalini et al. [33] analyzed three distinct points to evaluate the differences between strip sections. The results showed differences in the metabolites extracted in each section, suggesting the existence of a gradient in the length of the strip as some components are absorbed faster than others. Thus, for greater metabolome coverage, it is necessary to use the entire strip. Pure solvents, such as methanol [33,34,35,36] and acetonitrile [29], are frequently used in tear extraction after collection via TSs and capillary tubes. For AH and VH extraction, a single protein precipitation step can be applied using cold (below 4 °C) polar organic solvents, such as methanol 75–100% [37,38,39,40] and mixtures, including acetonitrile/methanol (1:1, *v*/*v*) [41,42,43,44,45,46], methanol/ethanol (1:1, *v*/*v*) [47], and chloroform/methanol (2:1, *v*/*v*) [31,48,49], are applied.

The investigation of biomarkers from the AH and VH provides a deeper understanding of ocular diseases, including Glaucoma [50], Congenital Ectopia Lentis [51], and Diabetic Retinopathy [52,53]. Nonetheless, tears have been the non-invasive biological specimen of choice to find potential biomarkers in systemic diseases such as Type 2 diabetes mellitus (T2DM) [36,54], and ocular disorders (Keratoconjunctivitis [55] and Keratoconus [56]). Brunmair et al. [36] demonstrated a significant increase in amino acid, carnitine, and uric acid levels in patients with T2DM. These metabolites have already been correlated with diabetes in metabolomics studies using other biofluids. Thus, the application of tears proved to be quite interesting and a less invasive alternative for diagnostics.

### 3.3. Sebum

Sebum is a complex mixture of oily and waxy lipids produced by the sebaceous glands that coat, hydrate, lubricate, thermoregulate, and protect the skin and hair. The sebaceous gland density is most prominent at the forehead, nose, and “t-zone” of the face. Sebum is mainly composed of glycerolipids (30–50%), free fatty acids (15–30%), cholesterol (1.5–2.5%), cholesterol esters (3–6%), squalene (12–20%), and wax esters (26–30%) [57]. The sebum composition may vary depending on the function in which it is involved [58]. The epidermis lipid surface in humans is uniquely composed of squalene and wax esters, not found anywhere else in the body. The metabolic pathways regulating its composition and secretion rate are still not understood; however, changes in sebum secretion rates have already been associated with some pathophysiologies such as acne and Parkinson’s disease [57,59,60]. Due to the interface between sebum and blood circulation, this specimen has potential for biomarker discovery. In this context, perillic aldehyde, hippuric acid, eicosane, and octadecanal were highlighted as volatile biomarkers from sebum samples in Parkinson’s patients, allowing the development of non-invasive patient screening methods [61]. Sebum sampling was recently explored to evaluate changes in the sebum lipid profiles of COVID-19 patients. After collection on the upper back using gauze and sample extraction with methanol, untargeted LC–MS analysis revealed that COVID-19 patients had reduced levels of triglycerides and ceramides compared to non-COVID-19 [62] subjects.

Developing sebum-based metabolomics (“sebomics”) involves the sampling, identification, and quantification of metabolites found in human sebum [59,63]. Multiple non-invasive sampling techniques have been developed, but no standardized approach exists. The intervariability of the skin surface and the sebum production rates of each individual presents a considerable challenge for sample collection, as this results in an uncontrollable measurement of the sample volume [59]. The most popular sampling technique is the application of adhesives, such as Sebutape^®^ (CuDerm Corp., Dallas, TX, USA) [64] or gauze [65], on the back or forehead. After collection, the sample must be sealed in background-inert plastic bags and stored at −80 °C. 

Dutkiewicz et al. (2017) developed an agarose hydrogel micropatch sorbent for sampling skin polar metabolites [66]. The authors combined it with a Nanospray Desorption Electrospray Ionization (nanoDESI)-MS as a non-invasive collection of skin excretion specimens to improve future diagnostic procedures. Using a medical-grade swab, the composition of sebum volatile organic compounds (VOCs) from the skin was recently explored by Zhang and collaborators [67] for biomarker discovery in different medical conditions, including T2DM and malaria. After sample collection, for the selective extraction of nonpolar metabolites, biphasic extraction protocols based on chloroform/methanol/water [68,69] or isopropanol/methanol mixtures [70] can be adopted. However, for comprehensive metabolite extraction, monophasic extraction protocols have been prioritized, using isopropanol, methanol, or ethanol to reduce toxicity and costs [71,72].

Generally, the sebum sampler is placed in a glass bottle for sebum extraction, and some additives, such as butylhydroxytoluene (BHT), can be added to the extraction solvent to prevent oxidation [64]. After vortex-mixing and sonication at room temperature, the extract is concentrated and stored at −80 °C until reconstituted for analysis. The reconstituted sample can be analyzed through LC–MS [73] or GC–MS [74].

### 3.4. Seminal Plasma

Seminal plasma consists of more than 95% of human semen and comprises secretions derived from the testicular, epididymis, and secondary sex glands [75]. It is a complex fluid that acts as a vehicle for transporting ejaculated spermatozoa from the testes to their destination. Seminal plasma contains a variety of proteins, ions, and metabolites, such as nucleosides, lipids, monosaccharides, amino acids, and steroid hormones [76]. It is the biological sample of choice for studying infertility [77,78,79,80], including the effects of exposure to toxic metals [81], or nicotine action [82] on sperm quality.

Seminal plasma can be obtained from the centrifugation of semen collected in universal containers for sampling through masturbation after 2–7 days of sexual abstinence. This collection is usually performed at reference centers. The frozen material, stored at −80 °C, is transported under refrigeration at low temperatures to a specialized laboratory to evaluate the ejaculate according to World Health Organization (WHO) criteria [83,84], followed by metabolite extraction.

A density gradient centrifugation at 600× *g* at room temperature (22 °C) for 20 min can be performed for separating spermatozoa from the semen liquid part [79,82,85] as it avoids damaging the cells. Spermatozoa must be lyzed using an established protocol to liberate all metabolites, and a detailed description can be found elsewhere [86].

The primary sample preparation protocol for seminal plasma is the solvent extraction–protein precipitation method. Usually, this procedure is performed using a mixture of water with highly polar organic solvents (1:1, *v*/*v*), such as methanol and/or acetonitrile, for polar metabolite screening [77,78,79,82,87]. In contrast, a mixture of methanol/chloroform/water (2:1:1, *v*/*v*/*v*) is often employed for lipid extraction [88]. After following the deproteinization method, a centrifugation step under refrigeration is recommended to remove any solid debris [79,81,87,89], followed or not followed by a filtration step using a nylon filter [78,81,89].

Although many metabolites have been reported in seminal plasma using the extraction protocol mentioned above and analysis using mass spectrometry, another less common protocol has also been proposed to improve the detection coverage of some functional groups. This method uses mixed-mode (reversed-phase and anion-exchange) SPE sorbents followed by chemical derivatization using pyridine to tag alcohols and carboxylic acid groups in seminal plasma [88]. Using this method, Wu and colleagues [88] found 624 molecular features in seminal plasma compared to 430 obtained with the classic solvent extraction–protein precipitation method. Xu and coworkers [80] applied the same protocol to evaluate infertility using a multi-analytical platform to cover polar and nonpolar metabolomes. However, it is important to note that the chemical derivatization procedure is functional-group-dependent. Furthermore, it increases both the cost and total time of sample analysis.

## 4. Underexplored Specimens in Clinical Metabolomics

### 4.1. Cerebrospinal Fluid (CSF)

The cerebrospinal fluid (CSF) is a clear fluid found within the brain’s ventricles in the subarachnoid spaces of the cranium and spine in all vertebrates. In addition to protecting the brain, the CSF has the vital functions of nourishment, the removal of degradation products of cellular metabolism, and preventing the accumulation of toxic levels of soluble metabolites such as hormones, neurotransmitters, and others throughout the Central Nervous System (CNS) [90] Until present, the Human Metabolome Database (HMDB) project [91] listed 468 small molecules in the metabolite catalog found in the human CSF. In addition to small metabolites, it is composed mainly of water and enzymes. The most identified metabolites are neurotransmitters, amino acids, carbohydrates, short-chain fatty acids, and alcohols, as well as metal ions and salts [92,93,94].

The metabolites can migrate from the blood to the CSF through the blood–brain barrier (BBB), affecting the brain cells and the function of the CNS [93]. Thus, the CSF is a key biological specimen for analyzing brain metabolism and understanding CNS diseases, providing insights into disease mechanisms [95]. Due to the increasing number of patients diagnosed with neurodegenerative and mental disorders such as multiple sclerosis [96], Parkinson’s disease [97,98], Alzheimer’s disease [99], epilepsy [100], and other slowly progressive diseases, the interest in the study of the CSF has expanded [93,101,102,103].

Unlike the other biological specimens discussed in this review, CSF sample collection is invasive, performed through lumbar puncture (LP), also known as a spinal tap. The patient is placed in the lateral recumbent position or remains sitting and leaning forward, and a sterile spinal needle is carefully inserted between vertebrae into the subarachnoid space at L3 /4 or L4/5 [103]. The volume of the CSF sampled is around 2.0–5.0 mL, and after centrifugation, the supernatant is stored at −80 °C [93]. Compared to plasma or urine sampling, CSF collection is so invasive that it requires highly trained personnel, making population studies difficult [104]. In this context, the metabolomics approach is occupying more space in CSF clinical analysis since detecting multiple metabolites in a single injection may reduce the number of sample collections for the same patient [105,106].

Untargeted metabolomics analysis via LC–MS is the most common strategy [105,107]. Whether the metabolomic analysis is for medium or highly polar metabolites, sample preparation generally consists of a single step of the protein precipitation protocol using methanol [95,108,109,110] or acetonitrile [111,112], or a 1:1 ratio of the two solvents [113], followed by centrifugation and extract concentration [114]. Lipid analysis performed using LC–MS after the B&D extraction method [69] was applied by Simone Bohnert et al. [115] as an innovative investigative method for CSF postmortem samples to aid interpreting death circumstances in the forensic context.

The volatile metabolome of CSF samples has also been investigated. A nonselective sample preparation approach applying LLE using methanol for protein precipitation and two-step derivatization using oximation and silylation was used in some studies, allowing the detection and quantification of different chemical classes through GC–MS [93,116]. Short-chain fatty acids (SCFAs) are a class of volatile metabolites that were extensively studied in the CSF once they were speculated to play a pivotal role in microbiota–gut–brain crosstalk [117]. The SCFAs butyrate and propionate have protective functions on the blood–CSF barrier. [92,108,118,119]. Typically, a SCFA is analyzed through GC–MS after a derivatization process [120] using a nonpolar (5%-phenyl)-methylpolysiloxane column and quantified in the selected ion monitoring (SIM) mode; however, methods without derivatization using polar stationary phases, such as the polyethylene glycol (PEG) column, are also described [121,122]. A GC–MS metabolomic approach of cerebrospinal fluid was evaluated in a naturally occurring depressive (NOD) model in a non-human primate (cynomolgus monkey, *Macaca fascularis*) and showed 37 metabolites identified as discriminant between NOD and healthy groups. Among these, SCFAs like acetic acid, propanedioic acid, and butyric acid were found to be disturbed in the CSF in NOD primates [123]. SCFAs can also be found in cerebrospinal fluid (CSF), typically in the ranges of 0–171 μmol L^−1^ for acetate, 0–6 μmol L^−1^ for propionate, and 0–2.8 μmol L^−1^ for butyrate, where they might influence the growth and differentiation of neurons and synapses, inflammatory responses, development, and the preservation of CNS homeostasis [119].

### 4.2. Hair

The hair is a non-invasive and conveniently collected sample that can be stored for long periods at room temperature. This biological specimen is composed of keratin and other fibrous proteins (approximately 90%), melanins, lipids, minerals, and water. Its chemical composition has been widely used in toxicology studies, specifically in biomonitoring exposure to drugs, metals, and alcohol [124,125]. Due to the incorporation of blood biomarkers into the hair follicles and their accumulation and distribution during growth, the hair can reflect long-term exposures [126]. For this reason, this specimen can be a good alternative for metabolomic studies, providing a broader view of the metabolic profile. Thus, in the last five years, hair samples have been widely used to study pregnancy complications, such as pre-eclampsia [127], fetal growth restriction [128], gestational T2DM [129], and diet restrictions [130]. Additional clinical investigations include the search for biomarkers in cervical cancer [131] and Alzheimer’s disease [132,133] and the monitoring of chronic conditions such as androgenetic alopecia [134] and baldness [135].

As a solid matrix in which the metabolites are deeply embedded, some care and the development of standard procedures are required for hair sample collection and preparation. The Society of Hair Testing (SoHT) provides protocols to achieve this standardization, including cutting, segmentation, decontamination, and homogenization steps before extraction. In summary, hair samples should be collected by cutting as close to the skin as possible and stored dry in the darkness at room temperature [136].

It is known that hair thickness varies depending on its location on the head. Such differences directly influence the absorption of species and chemical products and demonstrate differences in metabolic profiles. Thus, careful sampling is recommended, including different areas of the head for sample collection. Another important factor is the difference in capillary pigmentation arising from the genotype and phenotype. Different hair care practices and exposure to sunlight, wind, and humidity will directly affect hair’s metabolome and must be considered during biological interpretations of the results [137]. The segmentation through cutting between 10 and 30 mm is an optional step and provides historical insights into sample usage or exposures. A washing step, using water or organic solvents, serves as decontamination by removing cosmetic products, sweat, sebum, surface materials (such as skin cells, lice, and body fluids), or xenobiotic contaminants. After drying, the samples should be homogenized by being cut into small pieces or by going through pulverization or digestion methods [136].

A recent study employing LC–MS and GC–MS investigated pre-analytical factors and extractor solvents in the chemical profile of the hair. Reliable results and good metabolic coverage were achieved through successive decontamination using dichloromethane, acetone, water, and acetone followed by pulverization and extraction with acetonitrile/water [138].

Metabolite extraction is performed with or without alkaline hydrolysis, using NaOH 4 mol L^−1^ [139] or, more frequently, KOH 1 mol L^−1^, followed by neutralization with H_2_SO_4_ (3 mol L^−1^) [127,128,131]. Proteins are usually precipitated by the addition of pure methanol [125,129,134,135], methanol/phosphate saline buffer (PBS) [132,133,140], or methanol/water/acetone (1:1:1, *v*/*v*/*v*) [141]. In order to increase extraction performance, bench mills [142,143], ultrasound [132,133], and vortex [128,139] have been applied. The resulting supernatants can be directly analyzed through LC–MS, completely dried and resuspended in the respective mobile phases, or even derivatized for GC–MS analysis.

### 4.3. Saliva

Saliva is a biofluid secreted mainly by the parotid, submandibular, sublingual glands and several minor glands. It is a transparent liquid composed of water (99%), proteins/glycoproteins, oral bacteria, and blood cells. It is chemically composed of inorganic ions, lipids, hormones, and various metabolites, such as glucose, urea, some amino acids, hormones, and vitamins [144], varying in individuals according to gender and age. This biological fluid has multiple functions in the human body, emphasizing the lubrication of the oral cavity and aiding chewing and digestion, tooth protection, and defense against pathogens (viruses, bacteria, and fungi) [145,146].

Saliva reflects the healthy status of the human body since it contains biomarkers and blood-derived metabolism products [147] and can be used alternatively to plasma and serum. Despite having a comparable profile, the metabolites present in saliva are at lower concentrations than in blood. However, this has been a minor obstacle in metabolomics studies since the detection methods are highly sensitive [146]. Given the richness and metabolic response it provides, saliva samples have been used in screening for diagnosis and monitoring T2DM [148,149,150], neurological diseases (such as Alzheimer’s [151] and schizophrenia [152]), and some types of cancers (such as oral, breast, cerebral, colorectal, and gastric cancers) [153,154,155]. It is the ideal biofluid for studying oral diseases including periodontitis [156,157] and dental caries [158,159]. Recently, it has been applied to SARS-CoV-2 detection, diagnosing the coronavirus disease [160].

Saliva can be considered one of the easiest and cheapest biofluids to collect, with no risk to the individual, proving to be an alternative for children and patients with blood collection phobia, for example. Some important factors should be considered before sampling—time of the day for collection, drug and diet intake, smoking, and lifestyle—as they directly influence the metabolic profile of the samples [161]. Basic recommendations for pre-collection include not eating, drinking, smoking, or using medication at least 1–2 h before sampling. Additionally, some protocols require patient rest and the absence of oral-facial movements in the 5 min preceding the collection [162]. In terms of hygiene, most studies do not allow the application of oral hygiene products, in which the typical recommendation is washing the oral cavity with water [163,164,165,166,167]. Another important point is the type of saliva that will be used: stimulated or unstimulated. Some studies have shown that saliva stimulation causes variations in the metabolome, including some amino acid levels and metabolites from the urea cycle [168]. Stimulated saliva is obtained by chewing cotton or swab [169,170], paraffin wax [171,172,173], or even using chemical stimulants such as citric acid [174,175]. Such stimulus methods have been particularly important in obtaining samples from dehydrated patients with depression and chronic diseases with low salivary rates [175]. Unstimulated saliva, the most used in studies, is obtained by accumulating the fluid in the mouth, in which the sample can be collected by spitting [176,177,178] or through passive drooling [179,180]. Sample collection can be performed in sterile tubes through suction or using commercial device kits such as Salivette^®^ [181,182,183], Genotec^®^ [184], or Salimetrics^®^ [185,186]. Being a biofluid collected by the patient himself, the temperature, time of storage, and transportation are important factors that can affect the quality of the results. There is no consensus on the best condition, and some researchers recommend storage at 4 °C for a maximum of 6 h or maintenance at room temperature for 30–90 min [187,188]. However, keeping the samples at −20 °C or below as soon as possible after collection is recommended. Other pre-treatment procedures include centrifugation to remove cells and food debris followed by freezing storage (−80 °C).

The preparation of saliva samples for metabolomics analysis is quite simple. In general, the procedures involve protein precipitation with acetonitrile [189,190,191,192,193,194,195], methanol [196,197,198], or a combination of methanol/acetonitrile (1:1, *v*/*v*) [199,200,201], methanol/water [202,203,204,205,206], and methanol/acetonitrile/water (2:2:1, *v*/*v*/*v*) [207]. Other studies have used isopropanol [208] or acetone [209], which requires evaporation and resuspension in an appropriate solvent before chromatographic analysis. When GC–MS is used, the dried extracts are derivatized using oximation followed by a silylation step [209,210,211,212,213,214,215,216]. Methods involving headspace [217] and solid-phase microextraction (SPME) for VOC analysis [218] are also found.

### 4.4. Sweat

Human sweat has become a promising biological fluid in metabolomic studies, offering an opportunity to analyze the molecular composition of the body. It is secreted by apocrine, eccrine, and sebaceous glands and plays roles in the regulation of body temperature, the protection and hydration of the skin, and body homeostatic control [219]. It is a non-invasively collectible sample composed of metabolites, proteins, nucleic acids, and small particles like extracellular vesicles [220]. This biological specimen has been used in doping control testing, the exposure evaluation of heavy metals, and the routine diagnosis of cystic fibrosis (CF) [221]. In metabolomics studies, sweat samples can provide insights into health status and have been used to investigate autoimmune disorders [222,223] and T2DM [220] and for monitoring treatment efficacy for CF [224].

Sweat can be collected from different body regions, such as legs, arms (armpit and forearm), back, and fingers. A skin preparation is necessary to ensure sample purity by cleaning the area with alcohol and water. In general, sweat is stimulated by heat, exercise, or chemicals. By heating, the individuals are exposed to high temperatures (above 35–40 °C) or remain in a sauna. The chemical stimulation is achieved by applying pilocarpine to the skin followed by a low-intensity electrical discharge (3.0 mA for 5 min or less) [221]. The sample is collected in either its dry or fresh form, using or not using commercial devices.

Dry collection involves capturing the sweat on a solid support, like a gauze or filter paper, saturated with ethanol or a saline buffer. The sample is subsequently eluted and processed for analysis. Fresh sweat is collected from the skin using a pipette or directly into sterile tubes. Some protocols include a centrifugation, or fast filtration step to remove impurities, and lyophilization. The dry method is less invasive and offers a more standardized collection approach than the fresh collection. The samples are finally stored at −80 °C until analysis.

Sweat samples are usually extracted through protein precipitation using methanol [224], 50% acetonitrile [225,226,227], formic acid aqueous solution [228,229], or the mixture methanol/acetonitrile (1:1, *v*/*v*) [220] for nonpolar and polar metabolite coverage through LC–MS. After protein removal, a second extraction with dichloromethane was performed by Delgado-Povedano and collaborators [230], in which the organic phase was derivatized to evaluate the differences in metabolic profile between fresh and dry sweat-sample collection.

## 5. Conclusions

Although researchers are concerned about collecting and preparing biological specimens, the development of protocols and systematic evaluations of the procedures applied in metabolomics studies are scarce. This becomes more evident regarding biological specimens other than serum/plasma and urine, such as cerebrospinal fluid, hair, human breast milk, ocular fluids, saliva, sebum, seminal plasma, and sweat, considered in this review. The variable concentration range and physicochemical diversity of metabolites in complex organisms require well-designed methods to obtain reliable and high-quality data, which are crucial for biological interpretation. In this sense, it is important to emphasize the difficulty of estimating the number and dynamic range of the metabolites detected in the biological specimens revised here, whether due to the lack of identification of the metabolites, the type of analytical platform applied, or the lack of absolute quantification. Interesting information about the concentration ranges and patient status can be found for CSF, HBM, saliva, and sweat on the Human Metabolome Database (HMDB) platform (https://hmdb.ca/, accessed on 2 January 2024). Therefore, this review highlighted critical pre-collection factors of different biological specimens, considering non- or slightly invasive collection methods, such as saliva, tears, HBM, seminal plasma, sebum, sweat, and hair, which require the washing or disinfection of the area, as well as the choice of collection method and location. Also, highly invasive collection methods such as lumbar puncture and surgical collection for CSF and the AH/VH, respectively require highly qualified professionals. The samples should generally be stored and transported at low temperatures to avoid enzymatic activity and metabolite degradation. Extraction procedures involve LLE methods, with protein precipitation, followed or not followed by chemical derivatization, guided by the analytical platform of choice. The specimens reviewed here have demonstrated potential application in the study of chronic diseases, cancer, and degenerative diseases (such as Parkinson’s and Alzheimer’s) with a focus on discovering biomarkers and clinical diagnosis as they reflect the metabolic status of living organisms. They may be attractive alternatives for future investigations in clinical metabolomics.

## Figures and Tables

**Table 1:** Typical factors to be considered in a metabolomics study from pre-collection to metabolite extraction of clinical biological specimens.

Biological Specimen	Pre-Collection	Collection/Pre-Processing	Extraction
Cerebrospinal Fluid	Patient Position	Lumbar punctual	Protein precipitation
		Storage at low T	
Hair	Thickness Pigmentation	Cutting/segmentation	
		Decontamination	Alkaline hydrolysis
		Homogenization	Salt and protein removal
		Drying	Mechanical apparatus
		Storage at room T	
Human Breast Milk	Breast skin washing with water and soap Discard first drops	Manual or pump collection Pasteurization Storage at low T	Protein precipitation Dilution
Ocular Fluids	Ocular surface disinfection	Tears: absorbent materials or capillary collection	Mechanical devices (for absorbent collection) Protein precipitation
		AH and VH: surgical collection	
		Storage at low T	
Saliva	Food/drug intake	Stimulated vs. unstimulated	
	Drinking/smoking	Spitting, passive drool, or suction	Cell/food debris removal
	Oral hygiene products	Collection using commercial device kit	Protein precipitation
	Oral-facial movements	Storage at low T	
Sebum	Collection in the upper back or central region of the forehead	Adhesives or gauzes Storage at low T	Additives to prevent oxidation
			Protein precipitation
Seminal Plasma	Sexual abstinence	Slow centrifugation	Protein precipitation
		Storage at low T	
Sweat	Cleaning of collection area	Stimulation	Protein precipitation
		Dry or fresh collection	
		Filtration/lyophilization Storage at low T	

T, temperature; AH, aqueous humor; VH, vitreous humor. Details and direct link to the references are compiled in Table S1 of Supplementary Materials.

## Supplementary Materials

The following supporting information can be downloaded at: https://www.mdpi.com/article/10.3390/metabo14010036/s1, Table S1: Details of pre-collection, collection, and extraction conditions of uncommon and underexplored specimens in clinical metabolomics studies.
